# Novel culture media enhances mononuclear cells from patients with chronic limb-threatening ischemia to increase vasculogenesis and anti-inflammatory effect

**DOI:** 10.1186/s13287-021-02592-3

**Published:** 2021-09-28

**Authors:** Nuttapol Chruewkamlow, Kanin Pruekprasert, Phakawan Phutthakunphithak, Onchira Acharayothin, Tossapol Prapassaro, Kiattisak Hongku, Suteekhanit Hahtapornsawan, Nattawut Puangpunngam, Khamin Chinsakchai, Chumpol Wongwanit, Chanean Ruangsetakit, Nuttawut Sermsathanasawadi

**Affiliations:** 1 Research Department, Faculty of Medicine Siriraj Hospital, Mahidol University, Bangkok, Thailand; 2 Division of Vascular Surgery, Department of Surgery, Faculty of Medicine Siriraj Hospital, Mahidol University, 2 Wanglang Road, Bangkoknoi, Bangkok, 10700 Thailand

**Keywords:** Novel culture media, Mononuclear cells, Patients, Chronic limb-threatening ischemia, Vasculogenesis, Anti-inflammatory effect

## Abstract

**Background:**

Quality and Quantity culture media (QQ culture media) was reported to enhance vasculogenesis and angiogenesis function of mononuclear cells (MNCs) from healthy volunteers. In this study, MNCs from chronic limb-threatening ischemia (CLTI) patients were cultured in QQ culture media, and then investigated for angiogenesis-related phenotype and function.

**Methods:**

Patients aged ≥ 18 years with CLTI caused by atherosclerosis of the lower extremities were prospectively recruited at Siriraj Hospital (Bangkok, Thailand) during July 2017–December 2018. Peripheral blood mononuclear cells (PBMNCs) were isolated from peripheral blood. PBMNCs were cultured in either QQ culture media or standard culture media. The number of CD34+CD133+ cells, CD206+ cells, CD4+CD25+CD127+ cells, colony formation assay, and human umbilical vein endothelial cell (HUVEC) tube formation assay in MNCs were compared between those cultured in QQ culture media and those cultured in standard culture media.

**Results:**

Thirty-nine patients were included with a mean age of 69 ± 11 years. Diabetes mellitus was found in 25 (64%) patients. The percentage of CD34+CD133+ progenitor cells in MNCs cultured in QQ culture media and in MNCs cultured in standard culture media was 4.91 ± 5.30% and 0.40 ± 0.46%, respectively (*p* < 0.0001). The percentage of CD206+ cells in MNCs cultured in QQ culture media and in MNCs cultured in standard culture media was 19.31 ± 11.42% and 4.40 ± 2.54%, respectively (*p* < 0.0001). The percentage of inactive population of T regulatory cells (CD4+CD25+CD127+ cells) in MNCs cultured in standard culture media and in MNCs cultured in QQ culture media was 14.5 ± 10.68% and 1.84 ± 1.37%, respectively (*p* < 0.0001). The total number of colony-forming units from MNCs cultured in QQ culture media and in MNCs cultured in standard culture media was 8.86 ± 8.35 of 2 × 10^5^ cells/dish, and 0.58 ± 1.05 of 2 × 10^5^ cells/dish, respectively (*p* < 0.0001). The mean intensity of Dil-Ac-LDL uptake that incorporated into the HUVEC forming tube was 1.37 ± 0.88 in MNCs cultured in QQ culture media, and 0.78 ± 0.41 in MNCs cultured in standard culture media. (*p* < 0.0003).

**Conclusions:**

MNCs from CLTI patients that were cultured in QQ culture media had a significantly higher number of CD34+CD133+ cells and anti-inflammatory cells, and higher angiogenesis-related function compared to MNCs cultured in standard culture media.

## Introduction

Chronic limb-threatening ischemia (CLTI), which is an advanced stage of peripheral arterial disease, is characterized by severe occlusion of the arteries that markedly reduces blood flow to the lower extremities [1]. Symptoms and signs of CLTI include pain at rest, non-healing ulcer, and tissue gangrene leading to limb amputation—all of which are associated with high morbidity and mortality [1].

Endothelial progenitor cells (EPCs) were isolated and shown to be effective for promoting angiogenesis both in vitro and in vivo [2]. Several studies showed cell-based therapy using bone marrow or peripheral blood mononuclear cells (PBMNCs) to promote vascular angiogenesis to be safe and effective [3–6]. However, the number of EPCs in bone marrow and in PBMNCs is less than 0.01% and 0.1%, respectively [4, 7–10]. Moreover, the process of increasing the number of EPCs for effective therapeutic angiogenesis is time consuming and requires well-trained personnel [4, 7–10].

In 2014, Masuda et al. developed and reported a new culture media for MNCs that they named Quality and Quantity culture media (QQ culture media) [11]. QQ culture media was shown to enhance vasculogenesis and angiogenesis function of MNCs from healthy volunteers [11]. The MNCs cultured in QQ culture media (QQ-MNCs) showed higher therapeutic potential in vascular and tissue regeneration than the PBMNCs cultured in standard culture media [11, 12]. However, the effect of QQ culture media on MNCs from CLTI patients has not been investigated. In this study, PBMNCs from CLTI patients were cultured in QQ culture media, and then evaluated for their angiogenesis-related phenotype and function. Those results were then compared with those of PBMNCs cultured in standard culture medium.

## Materials and methods

### Patients

All patients aged 18 years or older with CLTI caused by atherosclerosis of the lower extremities who attended the CLTI clinic of the Division of Vascular Surgery, Department of Surgery, Faculty of Medicine Siriraj Hospital, Mahidol University, Bangkok, Thailand during July 2017 to December 2018 were prospectively invited to join this study. To be eligible for inclusion, patient candidates had to have at least one of the following presenting symptoms: ischemic rest pain, non-healing ulcer, or gangrene of lower extremity. Patients having other causes of CLTI, such as thromboangiitis obliterans, autoimmune disease, or thrombosed aneurysm, were excluded. Patients with severe infection and those not willing to join the study were also excluded. Patient demographic and clinical data, including age, gender, body mass index (BMI), comorbidity, and medical history of CLTI, were collected and recorded.

The study protocol was approved by the Ethical Review Committee of the Siriraj Institutional Review Board (COA No. 207/2560 [EC4]), and written informed consent was obtained from all patients.

### Cell culture

Fifteen milliliters (ml) of peripheral blood were collected by venous puncture of superficial vein at forearm. PBMNCs were isolated by density gradient centrifugation using Lymphocyte Separation Solution (Sigma-Aldrich Corporation, St. Louis, MO, USA). PBMNCs at a concentration at 2 × 10^6^ cells/2 ml were cultured either in QQ culture media [11] or in standard culture media [11] The cells were cultured in a 6-well Primaria dish (BD Biosciences, San Jose, CA, USA) for 7 days [11, 13].

### QQ culture media

The components of QQ culture media include the following: Stem Line II Solution (#S0192; Sigma-Aldrich) supplemented with five recombinant human proteins, including 100 ng/m of stem cell factor (SCF) (cat. no. #300-07; PeproTech, Cranbury, NJ, USA), 20 ng/ml of thrombopoietin (TPO) (#300-18; PeproTech), 100 ng/ml of Flt-3 ligand (#300-19; PeproTech), 50 ng/ml of vascular endothelial growth factor (VEGF) (#100-20; PeproTech), and 20 ng/ml of interleukin (IL)-6 (#200-06; PeproTech) [11].

### Standard culture media

Standard culture media is composed of 10% fetal bovine serum (FBS) (Gibco; Thermo Fisher Scientific, Waltham, MA, USA) and Roswell Park Memorial Institute (RPMI)-1640 Medium (Gibco; Thermo Fisher Scientific) [11, 13].

### Phenotypic analysis of progenitor cells, T regulatory cells, and M2 macrophages

After 7 days of cell culture, the cultured cells were harvested and washed with 2% FBS and 0.02% NaN_3_ phosphate-buffered saline (PBS) 2 times. Cells suspended in 2 mmol/L of EDTA/0.2% BSA/ PBS buffer were incubated after the addition of 10 µl of FcR blocking reagent at 4 °C for 30 min. The cells were then stained with markers of progenitor cells (CD34+CD133+ cells), M2 macrophages (CD206+ cells), and inactivated regulatory T cells (CD4+CD25+CD127+ cells) using a combination of monoclonal antibodies. In this study, two separate panels were used for phenotypic analysis. The first panel was used for the phenotypic analysis of progenitor cells and M2 macrophages. Cells were incubated with 5 µl of each mAb at 4 °C for 30 min. The mAbs included CD34-FITC (#343504; BioLegend, San Diego, CA, USA), CD11c-PE (#371504; BioLegend), CD133-APC (#372806; BioLegend), CD3-PE-Cy7 (300420; BioLegend), CD206-APC-Cy7 (#321119; BioLegend), and CD11b-PerCP Cy5.5 (#101228; BioLegend). The cells were then washed with 2% FBS and 0.02% NaN_3_ phosphate-buffered saline (PBS) 2 times and fixed with 1% paraformaldehyde (Sigma-Aldrich) in PBS. The cells were assessed using a BD LSR Fortessa Flow Cytometer™ (BD Biosciences, San Jose, CA, USA) [11, 14].

The second panel was used for the phenotypic analysis of inactivated T regulatory (CD4+CD25+CD127+) cells. Cells were incubated with 5 µl of each mAb at 4 °C for 30 min. The mAbs included CD25-PE (#302606; BioLegend), CD127-APC (#351316; BioLegend), CD3-Pe-Cy7 (#300420; BioLegend), and CD4-APC-Cy7 (#300518; BioLegend) [11, 14]. All experiments were performed in triplicate. The numbers of CD34+CD133+ cells, CD206+ cells, and CD4+CD25+CD127+ cells in PBMNCs cultured in QQ culture media and PBMNCs cultured in standard culture media were then compared.

### Endothelial progenitor cell (EPC) colony formation assay (EPC-CFA)

PBMNCs were harvested at a concentration of 1 × 10^5^ cells/ml and resuspended with 30% FBS/PBS 200 µl. The following recombinant human cytokines were then added to the cells: human SCF (#300-07; PeproTech) at a concentration of 66.7 ng/ml; human VEGF (#100-20; PeproTech) at a concentration of 33.3 ng/ml; human IL3 (#200-03; PeproTech) at a concentration of 13.3 ng/ml; human IGF-1 (#100-11; PeproTech) at a concentration of 33.3 ng/ml; human FGF Basic (#100-18B; PeproTech) at a concentration of 33.3 ng/ml; and, human EGF (#100-15; PeproTech) at a concentration of 33.3 ng/ml**.** The cell mixture was resuspended with complete MethoCult™ media (#04236; STEMCELL Technologies, Inc., Vancouver, British Columbia, Canada) at a final volume of 2 ml, and then cultured in a 37 °C environment for 14 days [11]. Endothelial progenitor cell (EPC) colony forming cells (EPC-CFCs) were assessed under phase-contrast light microscopy (Eclipse TE300; Nikon Instruments, Tokyo, Japan). A colony was defined as the presence of at least 50 cells [15]. All experiments were performed in triplicate. The numbers of colonies of PBMNCs cultured in QQ culture media and in standard culture media were compared.

### Tube formation assay

PBMNCs were labeled with 20 µg/ml of acetylated low-density lipoprotein and 1,10-dioctadecyl-3,3,30,30-tetramethyl-indocarbocyanine perchlorate (Dil-Ac-LDL) (Biomedical Technologies, Inc., Stoughton, MA, USA) at a concentration of 4 × 10^4^ cells/500 µl for 30 min in a 37 °C CO_2_ incubator. The PBMNCs were centrifuged down at 400 g for 10 min followed by washing with 2% FBS/PBS and suspension in 2% FBS/PBS at a concentration of 1 × 10^3^ cells/50 µl. The labelled PBMNCs were then cocultured with human umbilical vein endothelial cells (HUVECs) at an MNC-to-HUVEC ratio of 1 × 10^3^-to-1.5 × 10^4^ cells in a final volume of 100 µl. The cell mixture was incubated in a 37 °C water bath, and then 100 µl of the cell mixture was transferred into a pre-coated Matrigel (thin coat method) 50 µl/well in 96-well plates and incubated at 37 °C in a CO_2_ incubator for 10 h. The assessment of tube formation was performed using a Nikon Ti-S Intensilight Ri1 NIS-D inverted fluorescence microscope (Nikon Instruments). All experiments were performed in triplicate. The intensity of fluorescence from incorporated labeled PBMNCs from QQ culture media and from standard culture media in HUVECs was compared [11].

### Statistical analysis

All statistical analyses were performed using Prism 6 software (GraphPad Software, Inc., San Diego, CA, USA). Student's t-test was used for phenotypic analysis of progenitor cells, T regulatory cells, and M2 macrophages, and to compare colony formation and tube formation between MNCs cultured in QQ culture media and MNCs cultured in standard culture media. A *p*-value less than 0.05 was considered to be statistically significant.

## Results

Thirty-nine patients with chronic CLTI caused by atherosclerosis of the lower extremities were included. The mean age of study patients was 69 ± 11 years. There were 20 female and 19 male patients. Diabetes mellitus was found in 25 (64%) patients, and 1 (2.5%) patient reported being a smoker. Hypertension was found in 25 (64%) patients, and dyslipidemia was identified in 26 (67%) patients. Rest pain was reported by 6 (15%) patients. Gangrene was identified in 25 (64%) patients, and non-healing ulcer was found in 8 (21%) patients. The average ankle-brachial index (ABI) was 1.03 ± 0.71.

### Percentage of CD34+CD133+ progenitor cells in PBMNCs

**Fig. 1:**
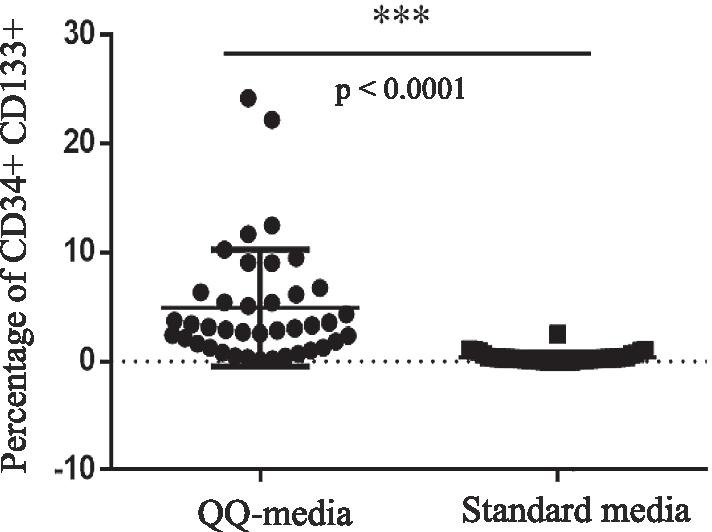
The percentages of CD34+CD133+ cells in peripheral blood mononuclear cells (PBMNCs) compared between PBMNCs cultured in Quality and Quantity culture media (QQ-media) and PBMNCs cultured in standard culture media. All experiments were performed in triplicate. Forward and side scatter plots were used to gate the entire population of mononuclear cells. The gated cells were then evaluated to identify CD3 and CD11c negative cells. The CD3 and CD11c negative cells were then gated to assess the population of CD34+CD133+ cells. Student's *t*-test was used for phenotypic analysis

The percentage of CD34+CD133+ progenitor cells was significantly higher in PBMNCs cultured in QQ culture media than in PBMNCs cultured in standard culture media (4.91 ± 5.30% *vs.* 0.40 ± 0.46%, *p* < 0.0001) (Fig. 1 and Table 1).

**Table 1:** Evaluated parameters compared between PBMNCs cultured in QQ culture media and PBMNCs cultured in standard culture media

Parameters	PBMNCs cultured in QQ culture media (mean ± SD)	PBMNCs cultured in standard culture media (mean ± SD)	*p*-value	Fold difference
CD34+CD133+ cells	4.91 ± 5.30%	0.40 ± 0.46%	< 0.0001	12.27-fold
CD206+ cells	19.31% ± 11.42%	4.40 ± 2.54%	< 0.0001	4.38-fold
CD4+CD25+CD127+ cells	1.84 ± 1.37%	14.5 ± 10.68%	< 0.0001	7.88-fold
CFU count (2 × 10^5^ cells/dish)	8.86 ± 8.35	0.58 ± 1.05	< 0.0001	15.27-fold
Dil-Ac-LDL uptake (reference intensity unit)	1.37 ± 0.88	0.78 ± 0.41	< 0.0003	1.71-fold

PBMNCs, peripheral blood mononuclear cells; QQ culture media, Quality and Quantity culture media; SD, standard deviation; CD, cluster of differentiation; CFU, colony-forming unit; Dil-Ac-LDL; Dil Acetylated Low-Density Lipoprotein

A *p*-value < 0.05 indicates statistical significance

### Percentage of CD206+ cells in PBMNCs

**Fig. 2:**
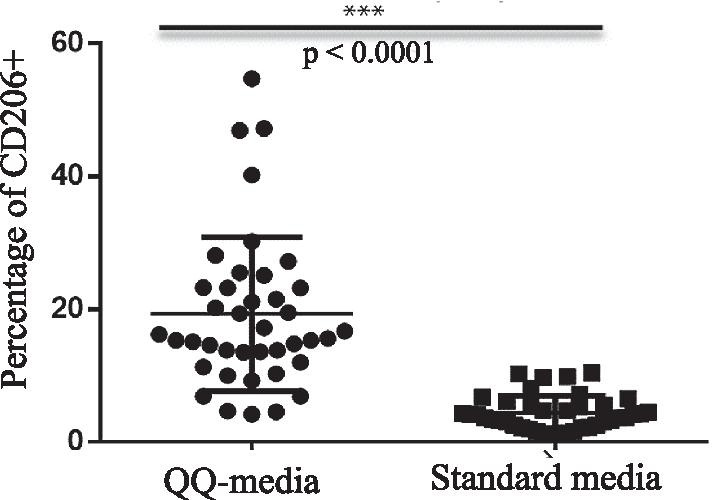
The percentages of CD206+ cells (M2 macrophages) in peripheral blood mononuclear cells (PBMNCs) compared between PBMNCs cultured in Quality and Quantity culture media (QQ-media) and PBMNCs cultured in standard culture media. All experiments were performed in triplicate. Forward and side scatter plots were used to gate the entire population of mononuclear cells. The gated cells were then evaluated to identify CD3 and CD11c negative cells. The CD3 and CD11c negative cells were then gated to assess the population of CD206+ cells. Student's *t*-test was used for phenotypic analysis

The percentage of CD206+ cells was significantly higher in PBMNCs cultured in QQ culture media than in PBMNCs cultured in standard culture media (19.31 ± 11.42% *vs.* 4.40 ± 2.54%, *p* < 0.0001) (Fig. 2, Table 1).

### Percentage of inactivated regulatory T cells (CD4+CD25+CD127+ cells) in PBMNCs

**Fig. 3:**
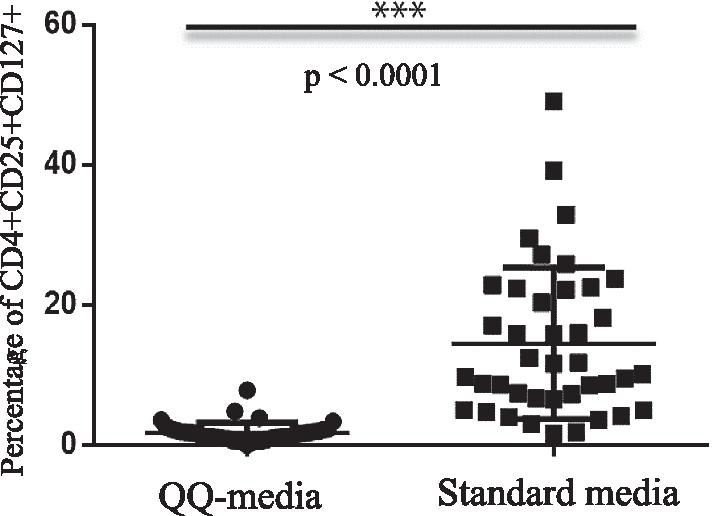
The percentages of CD4+CD25+CD127+ (inactivated T regulatory cells) in peripheral blood mononuclear cells (PBMNCs) compared between PBMNCs cultured in Quality and Quantity culture media (QQ-media) and PBMNCs cultured in standard culture media. All experiments were performed in triplicate. Forward and side scatter plots were used to gate the lymphocyte populations. The gated cells were then evaluated to identify CD3+CD4+ cells. The CD3+CD4+ cells were then gated to assess the populations of CD4+CD25+and CD127+ cells. Student's *t*-test was used for phenotypic analysis

The percentage of inactivated T regulatory cells (CD4+CD25+CD127+ cells) in PBMNCs cultured in standard culture media was 14.5 ± 10.68%. In contrast, the percentage of CD4+CD25+CD127+ cells in PBMNCs cultured in QQ culture media was 1.84 ± 1.37%. The percentage of inactivated T regulatory cells in PBMNCs cultured in QQ culture media was significantly lower (*p* < 0.001) (Fig. 3, Table 1). A lower number of inactivated regulatory T cells was considered to reflect the anti-inflammatory effect of QQ-MNCs.

### EPC-CFA in PBMNCs

**Fig. 4:**
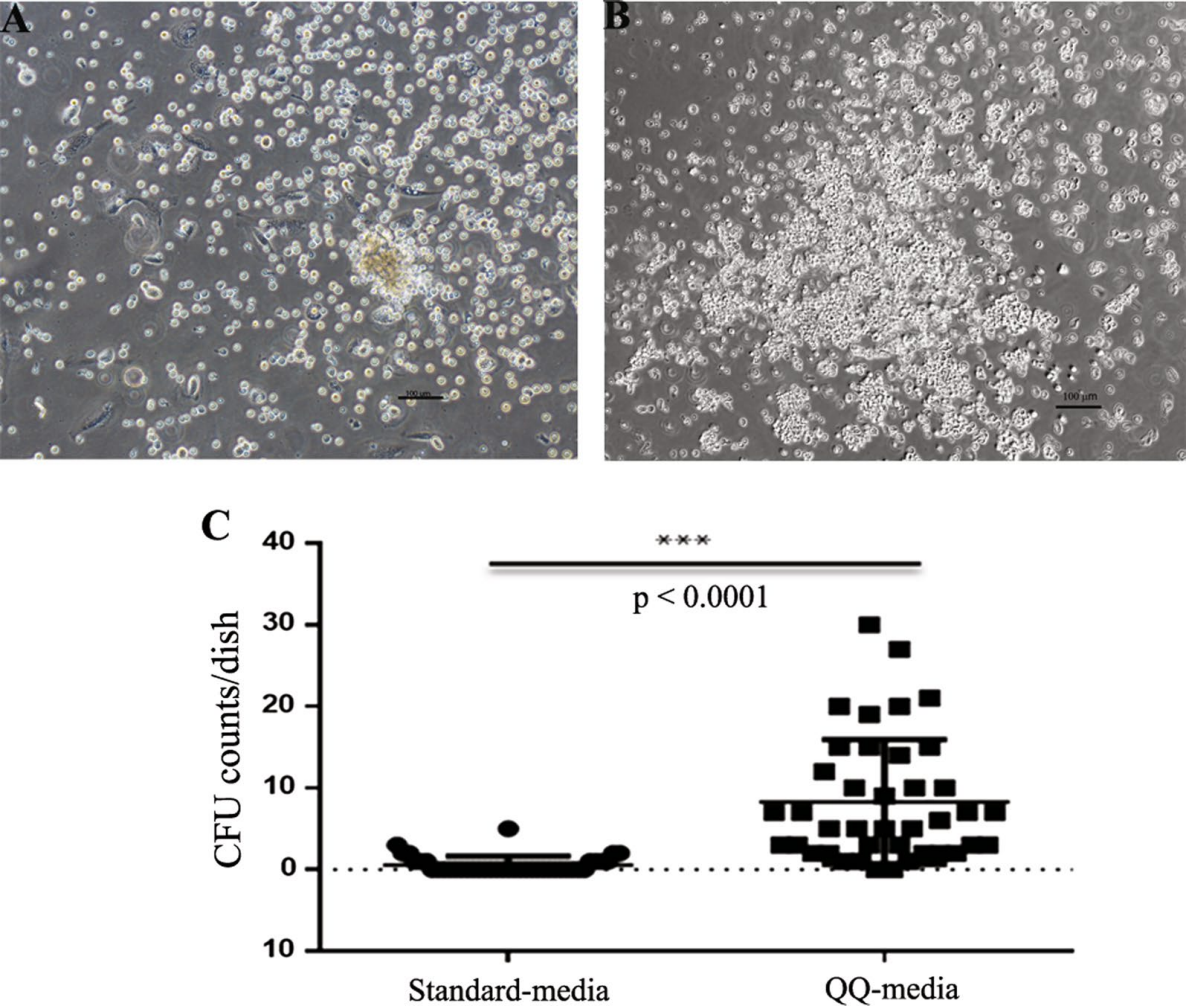
Colony formation assay. **a** Peripheral blood mononuclear cells (PBMNCs) cultured in standard media, **b**PBMNCs cultured in QQ-media, and **c** colony-forming unit counts per dish compared between PBMNCs cultured in QQ-media and PBMNCs cultured in standard culture media. Scale bar = 100 µm

The total number of CFUs from PBMNCs cultured in QQ culture media and PBMNCs cultured in standard culture media was 8.86 ± 8.35 of 2 × 10^5^ cells/dish and 0.58 ± 1.05 of 2 × 10^5^ cells/dish, respectively. The total number of CFUs in PBMNCs cultured in QQ culture media was significantly higher than the total number observed in standard culture media-cultured PBMNCs (*p* < 0.001) (Fig. 4 and Table 1).

### Tube formation assay in PBMNCs

**Fig. 5:**
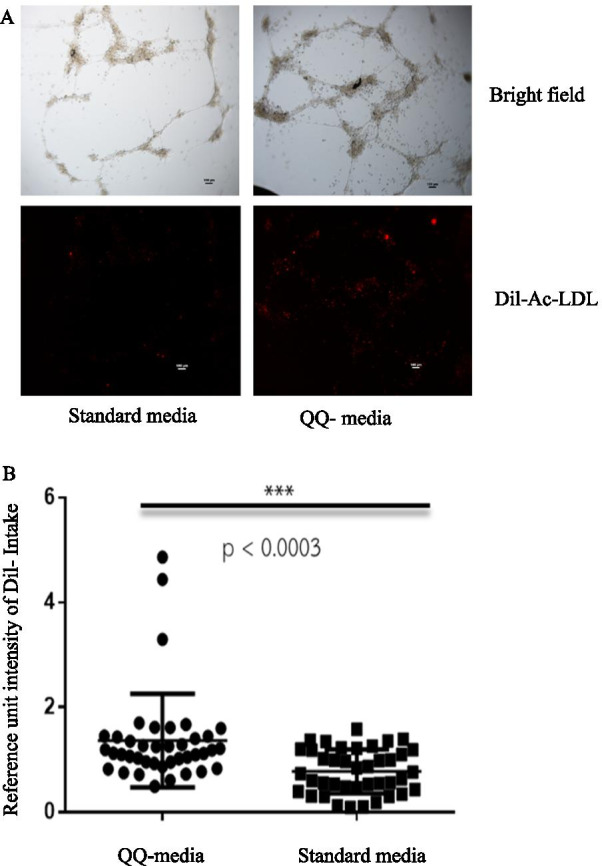
The results of tube formation assay compared between peripheral blood mononuclear cells (PBMNCs) cultured in Quality and Quantity culture media (QQ-media) and PBMNCs cultured in standard media. **A** Bright field and Dil-Ac-LDL images compared between standard culture medium-cultured PBMNCs (PBMNC) and QQ-media-cultured PBMNCs (QQMNC). **B** Reference unit intensity of Dil-Ac-LDL uptake compared between PBMNCs cultured in QQ-media and PBMNCs cultured in standard culture media. Scale bar = 100 µm

The mean intensity of Dil-Ac-LDL uptake that incorporated into the HUVEC forming tube was 1.37 ± 0.88 in PBMNCs cultured in QQ culture media, and 0.78 ± 0.41 in PBMNCs cultured in standard culture media. Dil-Ac-LDL uptake was significantly higher in PBMNCs cultured in QQ culture media. (*p* < 0.0003) (Fig. 5, Table 1).

## Discussion

In this study, we investigated the angiogenesis-related phenotype and function of PBMNCs from CLTI patients that were cultured in QQ culture media (QQ-MNCs), which was developed and published by Musuda et al. [11]. Our results showed that QQ culture media enhanced angiogenesis, yielded more stem cell progenitor cells, and increased the anti-inflammatory cell population—all compared to the results observed from PBMNCs cultured in standard culture media. Those observed characteristics suggest the potential of QQ-MNCs as a novel therapeutic approach for treating CLTI patients. Most patients with CLTI caused by atherosclerosis have impaired regenerative progenitor cell function [11, 16]. Moreover, diabetes mellitus type II leads to high inflammation and reduced regenerative capability of the vascular system [2, 7, 11, 12, 17].

Several studies have reported aging or older age status to be associated with very high titer of inflammatory cytokines, such as TNF-α and IFN-γ. Both of these cytokines can hinder the regenerative function of progenitor cells. The cause of high expression of inflammatory cytokines in older adults is impaired regulatory T cell function [18, 19].

In vitro and animal study QQ-MNCs from healthy patients demonstrated high vasculogenic conditioning [11]. In the present study, we studied QQ-MNCs from CLTI patients. Most patients in our study were aged > 70 years, and 63% of our patients had diabetes mellitus. PBMNCs from these CLTI patients demonstrated high vasculogenic conditioning after culturing in QQ culture media.

In CLTI patients, the number of CD34+CD133+ progenitor cells, which are considered to be vasculogenic progenitor cells that play an important role in angiogenesis, decreased [11, 20, 21]. In contrast, we found that QQ-MNCs from CLTI patients had a higher number of CD34+CD133+ cells. Masuda et al. reported that QQ-MNCs exhibited slightly increased kinase insert domain receptor (KDR)+ cells compared to PBMNCs [11]. However, they found the population of CD31+ cells in QQ-MNCs and PBMNCs to be comparable [11].

Previous study found cell populations of QQ-MNCs not only to be increased in CD34+ or CD133+ cell populations (which indicates an expanded population of immature EPCs), but also to be increased in CD105+ or CD146+ cell populations (which is indicative of EPC expansion and differentiation) [11]. These data suggest that QQ culture promotes EPC expansion and differentiation.The QQ-MNCs in our study also demonstrated a significantly greater number of M2 macrophages (CD206, anti-inflammatory macrophages), and significantly less inactivated regulatory T cells (CD4+CD25+CD127+ cells). The CD4+CD25+CD127± phenotype represents a T regulatory subset of T cells, and it plays an important role in controlling immune regulation, suppressing the immune system, and also in controlling the level of inflammatory cytokine release [11, 20]. A recent study reported a relationship between CD4+CD25+CD127± and FoxP3 level, which is considered to be immune suppression population of T regulatory cells [22, 23]. CD4+CD25+CD127+ cells were reported to be associated with low expression of FoxP3 in T regulatory cells, and they are considered to be inactive T regulatory cells that play a role in immune regulation [22, 23]. The results of our study showed the number of CD4+CD25+CD127+ cells to be lower in QQ-MNCs than in PBMNCs cultured in standard culture media. This finding suggests that QQ culture media could activate the regulatory function of T regulatory cells of PBMNCs, which effectuated the suppression of inflammation [11, 23, 24]. These findings suggest that QQ-MNCs yield increased numbers of active regulatory T cells, and that they promote the development of anti-inflammatory cell population, including M2 macrophages [11].

Moreover, QQ-MNCs from CLTI patients had high angiogenesis-related function as exhibited by colony formation assay and tube formation assay, as shown in Figs. 4 and 5, respectively. Previous study reported that EPC-CFCs were derived from CD34+ cells because CD34+ cell-depleted MNCs did not yield EPC colony; however, CD34+ MNCs after QQ culture contained EPC-CFCs [11]. So, the higher number CD34+ cells in QQ-MNCs in our study might be the cause of the higher number of EPC-CFCs from QQ-MNCs compared to the number coming from PBMNCs.

Although benefit of autologous intramuscular injection of MNCs in no-option CLTI has been reported, a recent meta-analysis did not show a clear benefit of mononuclear cell injection on clinical outcomes in CLTI patients [25, 26]. This could be due to the low quantity and low quality of MNCs from CLTI patients [27]. Our study showed that QQ-MNCs from CLTI patients had a significantly high number of CD34+CD133+ cells, a significantly higher number of anti-inflammatory cells, and significantly higher angiogenesis-related function compared to PBMNCs in in vitro study. The clinical benefit of QQ-MNCs should now be evaluated in in vivo study. As another option for treating CLTI patients with stem cells, some researchers are studying allogeneic mesenchymal stem cell (MSCs) for treating CLTI [28–30]. Nevertheless, future high-quality large clinical studies are needed to prove the efficacy of MSCs in the treatment of CLTI.

There are some limitations of this research. QQ-MNCs cultured in a HUVEC tube formation assay is a weak indicator of efficient vasculogenic power in vivo. Tube formation assay with CD34+/CD133+ cells from QQ-MNCs should be further studied in vitro, and further study of QQ-MNCs for cell-based therapy should be conducted in vivo using intramuscular injection of QQ-MNCs from CLTI patients into the limbs of mice whose arteries were ligated.

## Conclusions

QQ-MNCs from CLTI patients had a significantly higher number of CD34+CD133+ cells, a significantly higher number of anti-inflammatory cells, and significantly higher angiogenesis-related function compared to MNCs cultured in standard culture media as demonstrated by both colony formation unit assay and HUVEC tube formation assay.

## Data Availability

The data that support the findings of this study are available from the corresponding author upon reasonable request.

## Abbreviations

QQ: Quality and quantity
MNCs: Mononuclear cells
CLTI: Chronic limb-threatening ischemia
PBMNCs: Peripheral blood mononuclear cells
HUVEC: Human umbilical vein endothelial cell
EPCs: Endothelial progenitor cells
ml: Milliliter
ng: Nano gram
IL: Interleukin
FBS: Fetal bovine serum
PBS: Phosphate-buffered saline
FC: Fragment crystallizable
µl: Microliter
mAb: Monoclonal antibodies
Dil-Ac-LDL: Acetylated low-density lipoprotein and 1,10-dioctadecyl-3,3,30,30-tetramethyl-indocarbocyanine perchlorate
ABI: Average ankle-brachial index
IFN: Interferon
TNF: Tumor necrosis factor
